# Synchronous Periprosthetic Joint Infections: A Scoping Review of the Literature

**DOI:** 10.3390/diagnostics12081841

**Published:** 2022-07-30

**Authors:** Andrea Sambri, Emilia Caldari, Michele Fiore, Claudio Giannini, Matteo Filippini, Lorenzo Morante, Claudia Rondinella, Eleonora Zamparini, Sara Tedeschi, Pierluigi Viale, Massimiliano De Paolis

**Affiliations:** 1 Orthopaedic and Traumatology Unit, IRCCS Azienda Ospedaliera-Universitaria di Bologna, 40138 Bologna, Italy; emiliacaldari@gmail.com (E.C.); michele.fiore@ior.it (M.F.); claudio.giannini@ior.it (C.G.); matteo.filippini@ior.it (M.F.); lorenzo.morante@ior.it (L.M.); claudia.rondinella@ior.it (C.R.); massimiliano.depaolis@aosp.bo.it (M.D.P.); 2 Infectious Disease Unit, IRCCS Azienda Ospedaliera-Universitaria di Bologna, 40138 Bologna, Italy; eleonora.zamparini@aosp.bo.it (E.Z.); sara.tedeschi@aosp.bo.it (S.T.); pierluigi.viale@unibo.it (P.V.); 3 Department Medical and Surgical Sciences, DIMEC Alma Mater Studiorum, University of Bologna, 40126 Bologna, Italy

**Keywords:** prosthetic joint infection, synchronous infections, multiple infections

## Abstract

Prosthetic joint infections (PJIs) occurring in multiple joints at the same time (synchronous PJI) are an extremely rare complication, frequently associated with bacteremia, and are associated with high mortality rates. The presence of three or more prosthetic joints, rheumatoid arthritis, neoplasia, bacteremia and immune-modulating therapy seem to be the recurring risk factors for synchronous PJI. In case of PJIs, all other replaced joints should be considered as potentially infected and investigated if PJI is suspected. Treatments of synchronous multiple PJIs vary and must be decided on a case-by-case basis. However, the advantages of one-stage exchange seem to outweigh the two-stage protocol, as it decreases the number of necessary surgical procedures. Nonetheless, too few studies have been conducted to allow firm conclusions about the best handling of synchronous PJI. Thus, additional studies are needed to understand this devastating complication and to design the most appropriate diagnostic and therapeutic path.

## 1. Introduction

Prosthetic joint infections (PJIs) are a rare but serious complication following arthroplasties [1]. PJIs occurring in multiple joints represent an even rarer complication, frequently associated with bacteremia, associated with high mortality rates. Most of them are metachronous, occurring at a different time in multiple replaced joints [2].

The incidence of synchronous PJI, occurring simultaneously at different sites, is extremely low [3,4,5,6,7,8]. However, the increasing number of people carrying multiple arthroplasties in their bodies increases the population at risk for synchronous PJI.

In the literature, there is an overall paucity of data available on the clinical features and outcomes of patients with synchronic PJI, with most of the data being extrapolated from heterogeneous series that include metachronous and synchronous multiple PJIs [3,6].

The aim of this scoping review is to provide an overview on the main aspects synchronous multiple PJIs, such as epidemiology, risk factors and management, in order to find specific variables worthy of further investigation by specific studies.

## 2. Materials and Methods

**Figure 1:**
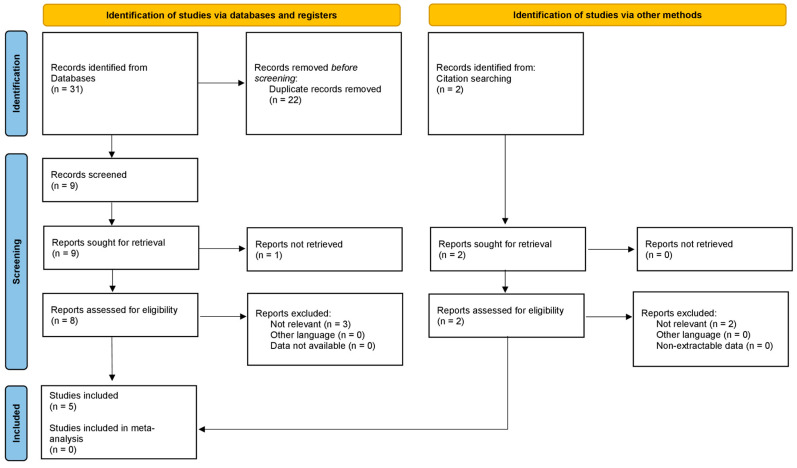
PRISMA ScR algorithm of the included studies.

An in-depth search of the scientific research was performed according to PRISMA (Preferred Reporting Items for Systematic Reviews and Meta-Analyses) Extension for Scoping Reviews (PRISMA ScR) [9]. The search algorithm according to these guidelines is shown in Figure 1.

A search regarding the existing evidence on synchronous periprosthetic joint infections with no restrictions on date of publication, up to the end of September 2021, was performed on the PubMed (https://pubmed.ncbi.nlm.nih.gov/ (accessed on 30 June 2022)), Scopus (https://www.scopus.com (accessed on 30 June 2022)), and Cochrane Library (https://www.cochranelibrary.com/ (accessed on 30 June 2022)) databases. Various combinations of the following keywords were used: “synchronous periprosthetic joint infection”, “multiple joint infection”, and “multiple arthroplasty infection”. The inclusion criteria were as follows: original research reporting clinical outcomes synchronous periprosthetic joint infections in the English language. The studies reporting data on multiple infections (both metachronous and synchronous) regardless of the time of infections’ onset were retained because of the presence of evidence of potential interest, but were not included in the main results of the research. The studies were categorized by study type, according to the Oxford Centre for Evidence-Based Medicine. We excluded animal studies, cadaveric studies, biomechanical reports, case reports, literature reviews, editorial articles, surgical technique descriptions, and instructional courses. Articles that were considered relevant during the electronic search were retrieved in full-tex formt, and a cross-referencing hand-search of their bibliographies was performed in order to find further related articles. Reviews and meta-analysis were also analyzed in order to broaden the search for studies that might have been missed through the electronic search.

The main clinical aspects evaluated in this review were epidemiology, risk factors, diagnosis, and treatment.

To assess the quality of the articles, the Institute of Health Economics (IHE) Quality Appraisal Checklist for Case Series Studies, which assesses methodologies based on 20 criteria (Table 1), was performed. The IHE checklist stratifies the quality of studies using a continuous scale of values. However, to facilitate the readability of the data, the authors considered it useful to artificially introduce a categorical stratification into (1) high quality studies, if the positive responses to the queries totaled >15; (2) moderate quality studies, if they totaled ≤15 and ≥10; (3) low quality studies, if they totaled <10. Each study was assessed by two reviewers (M.F. and C.G.) independently and in duplicate; disagreement was resolved by the senior author (M.DP.).

## 3. Results and Discussion

Only five studies reported series of synchronous or both synchronous and metachronous periprosthetic joint infections in which the data concerning synchronous infections were clearly distinguishable [4,5,6,7,8] (Table 1, Table 2, Table 3, Table 4 and Table 5). Three further studies were considered as reporting data on multiple PJIs [10,11,12] (Table 5). The overall quality of the series assessed via IHE checklist was found to be moderate in all the cases (Table 1).

### 3.1. Epidemiology

The incidence of synchronous PJI is low and not well studied. It is estimated to range between 1.4% and 5% of all PJIs (Table 2).

Zeller et al. [4] found 16 (1.4%) patients with synchronous PJI in a cohort of 1185 patients affected by PJIs. PJIs involved bilateral total hip arthroplasty (THA) in eight patients, bilateral total knee arthroplasty (TKA) in three patients, one THA and TKA in four patients. In one patient, a bilateral TKA, one THA and one toe arthroplasty were infected. (Table 3)

Gausden et al. [6] treated 2671 PJIs between 1990 and 2018, identifying only 34 patients (1.3%) who developed PJIs involving more than one joint simultaneously. PJIs involved bilateral TKA in 27 patients, bilateral THA in 3 patients, one TKA and one total shoulder arthroplasty in 1 patient, one TKA and one total elbow arthroplasty in 1 patient, and bilateral THA and one TKA in 1 patient.

Thiesen at al. [5] analyzed a selected cohort of 644 patients, and the incidence of synchronous PJI was as high as 4%. They found that 20 THA, 15 TKA and 7 total shoulder arthroplasties were implicated, but the distribution of these in the patients was not indicated.

Komnos et al. [7] found a higher prevalence of synchronous PJI (5%). They did not specify sites of synchronous PJI, but they indicated that 19 THA and 4 TKA were involved.

Abblitt at al. [8] considered 76 patients with multiple PJIs, 4 of whom had synchronous PJI. (5%) The PJIs involved were THA + TKA in one patient and bilateral TKA in three patients.

The bacteria associated with synchronous PJI are similar to those found in single-joint PJIs. *Staphylococcus aureus* (both methicillin sensible and resistant), *Streptococcus* and *Escherichia coli* are the most common causative organisms. Another frequently observed causative microorganism is *Staphylococcus epidermidis*, a low-virulence pathogen that causes slowly progressing infections [7,13] (Table 4).

Gausden at al. [6] reported an increased mortality rate in synchronous PJI (18% within 30 days and 27% within 1 year) compared to a single PJI (a 1-year mortality of 8% and a 5-year mortality of 21%) [14,15].

### 3.2. Risk Factors

Multiple etiological risk factors for PJIs have been proposed in the literature [16,17]. However, only sparse information has been reported on risk factors for synchronous multiple PJIs. (Table 5)

Thiesen et al. [5] identified the presence of three or more arthroplasties, rheumatoid arthritis, a history of neoplasia, the use of immune-modulating therapies and sepsis as risk factors for developing synchronous PJI.

Jafari et al. [10] reported that the risk of synchronous PJI is increased in immunocompromised patients.

Luessenhop et al. [11] identified rheumatoid arthritis as the leading risk factor for multiple PJI, although they did not distinguish between metachronous and synchronous PJI.

Zeller et al. [4] underlined that all synchronous PJIs were secondary to a hematogenous spread, occurring after a long infection-free interval. Bacteremia and a distant infectious focus were identifiable in half of the patients. In two patients of their series, the bacteremia source was an early postoperative infection of a prosthesis that subsequently spread to another prosthesis. In other patients of that series, distant foci were identified in an endovascular prosthesis, spondylodiscitis, and endocarditis of a pacemaker.

Haverstock et al. [12] reported that acute hematogenous spread was involved in approximately 50% of patients with multiple PJIs in their series.

Abblitt et al. [8] observed bacteremia as a significant risk factor for multiple PJIs.

Gausden et al. [6] identified acute infections caused by hematogenous spread and bacteremia in 41% of their patients.

### 3.3. Diagnosis

Different classification systems can be used for the diagnosis of PJI, including those reported by the Musculoskeletal Infection Society (MSIS) and those reported by the European Bone and Joint Infection Society (EBJIS) [18,19]. Clinical suspicion and a thorough history remain the basis of correct evaluation [20]. This is the first step in risk stratification and guides the strategy for the execution of subsequent diagnostic tests. A suspicious clinical presentation is defined as pain, heat, joint effusion, reddening and joint dysfunction. Nevertheless, the absence of clinical signs is not always indicative of the absence of infection. In the series by Thiensen et al. [5], only 14 (54%) out of 26 patients affected by a synchronous PJI showed clinical signs of infection in all infected joints.

Isolation of the causative microorganism from cultures of fluid or tissue obtained from the affected joint is critical for the selection of suitable antibiotic therapy and can supply information on prognosis. However, cultures are unfortunately limited by poor sensitivity and may remain negative in up to 20% of patients with underlying PJI [21]. This evidence is confirmed in the synchronous PJI. In the series by Gausden et al. [6], cultures were negative in 11 of 34 patients (32%). In most of the cases, the inability to isolate the infecting organism is due to the administration of antibiotics prior to obtaining fluid or tissue samples from the affected joint. Therefore, a 2-week antibiotic-free interval is suggested before obtaining culture samples [22].

Imaging is rarely diagnostic of infection and the role of nuclear medicine studies in the diagnosis of PJI is now much more limited than in the past. However, X-rays can identify periosteal new bone formation, which is considered a specific feature of PJIs, though with low sensitivity (16%) in early cases. Other signs of PJIs on X-rays include sclerosis, cortical thickening, soft tissue gas, and component loosening. TC and MRI are not normally useful for the diagnosis of PJIs. Regarding nuclear medicine, the initial radionuclide test made is generally bone scintigraphy; this has high sensitivity, but low specificity, but if it is negative the diagnosis of PJI can be excepted. In the case of positive bone scintigraphy, the addition of labeled leukocyte scintigraphy significantly increases the diagnostic reliability for PJIs [23]. Therefore, in the case of a confirmed PJI, all other replaced joints should be investigated.

In the suspicion of synchronous PJI, blood cultures for aerobic and anaerobic microorganisms are critical too, given that most authors agree that synchronous PJI is very often related to bacteremia.

In the case of PJI confirmed in one joint, it is still debated as to whether all replaced joints should be aspirated. Zeller et al. [4] and Wouthuyzen-Bakker et al. [24] did not routinely aspirate all joints with total joint arthroplasty in the case of a PJI. The choice to perform joint aspiration or not was based on clinical signs, symptoms, suspicious radiographs (periosteal reaction) or a case of sepsis. Similarly, Gausden et al. [6] did not routinely aspirate other joints when a patient presented with a single PJI, unless the other joints were symptomatic. On the other hand, Thiensen et al. [5] recommended performing the aspiration of all replaced joints if another site PJI was diagnosed. This is particularly suggested in the case of a suspected synchronous PJI caused by a low-virulence pathogen. Komnos et al. [7] reported on 10 patients who underwent aspiration of a joint other than the one with a confirmed PJI. Four of these aspirates were positive, with one of them being asymptomatic. Thus, the authors suggested that in a case of a confirmed PJI, an aspiration of the other joints with a prosthesis in place should be considered for patients presenting with risk factors for synchronous PJIs.

### 3.4. Treatment

Regarding the PJI treatment, the latest guidelines show that the medical–surgical management strategy has to be decided in multidisciplinary consensus meetings, composed of orthopedic surgeons and infectious disease specialists, guided by the type of PJI, the isolated microorganism and its antibiotic susceptibility [25,26].

Debridement and implant retention can be attempted in acute post-operative and hematogenous PJI occurring earlier than 30 days after prosthesis implantation or <3 weeks since the onset of symptoms and in the absence of a sinus tract [27]. A one-stage exchange strategy for the treatment of PJI may be considered in patients with an infection who have a good soft tissue envelope, provided that the pathogens are known preoperatively and that they are susceptible to oral antimicrobials with excellent oral bioavailability [28]. Two-stage exchange strategy is indicated in patients who are not candidates for a one-stage exchange, with pathogens which are unknown preoperatively or difficult to treat, who are medically able to undergo multiple surgeries [29].

The best treatment approach is not as clear when dealing with synchronous PJI, considering that very few data are available on the management of concomitant multiple PJIs. In their series, Zeller et al. [4] treated three patients affected by an acute PJI with simultaneous debridement and prosthesis retention of all affected joints. Six patients with a delayed or late PJI were treated with subsequent one-stage exchange arthroplasties with an interval of 40 days between the two operations. One patient was managed with a two-stage exchange of a hip prosthesis, followed by a one-stage exchange of the contralateral hip prosthesis. Another patient underwent permanent resection arthroplasty on the hip prosthesis, followed one month later by two-stage exchange of the other hip prosthesis. Palliative surgeries and suppressive antibiotic therapy were performed in very old patients (range 78–93 years) and in those with a high surgical risk. Palliative surgery included the drainage of an abscess in two patients with bilateral THA and bilateral TKA infections, one-stage exchange for only one loosened and painful prosthesis in one patient with bilateral THA infection, and resection of the toe arthroplasty in one patient with bilateral TKA + THA + toe arthroplasty infection. Gausden et al. [6] used debridement and implant retention in 23 patients, two-stage exchange of all involved joints in 10 patients and a combination of both approaches in 1 patient. Thiensen et al. [5] used a one-stage exchange of all affected joints at the same surgery.

## 4. Conclusions

Synchronous PJI is a rare but very serious complication associated with high patient mortality and a high risk of reinfection. The presence of three or more prosthetic joints, rheumatoid arthritis, neoplasia, bacteremia and immune-modulating therapy seems to be the recurring risk factors for synchronous PJI. In the case of PJI, all other replaced joints should be considered as potentially infected and investigated if PJI is suspected.

Treatment of synchronous multiple PJI is various and must be decided on a case-by-case basis. However, the advantages of one-stage exchange seem to outweigh those of the two-stage protocol, as it decreases the number of necessary surgical procedures. Nonetheless, too few studies have been conducted to allow firm conclusions about the best handling of synchronous PJI. Thus, additional studies are needed to understand this devastating complication and to design the most appropriate diagnostic and therapeutic path.

## Figures and Tables

**Table 1:** IHE quality appraisal checklist for case series included in this review.

Study	Zeller et al. [4]	Gausden et al. [5]	Thiensen et al. [6]	Komnos et al. [7]	Abblitt et al. [8]
Q1: was the hypothesis/aim/objective of the study clearly stated?	yes	yes	yes	yes	yes
Q2: was the study conducted prospectively?	no	no	no	no	no
Q3: were the cases collected in more than one centre?	no	no	no	no	no
Q4: were patients recruited consecutively?	yes	yes	yes	yes	yes
Q5: were the characteristics of the patients included in the study described?	yes	yes	yes	yes	yes
Q6: were the eligibility criteria (i.e., inclusion and exclusion criteria) for entry into the study clearly stated?	yes	yes	yes	yes	yes
Q7: did patients enter the study at a similar point in the disease?	yes	yes	yes	yes	yes
Q8: was the intervention of interest clearly described?	yes	yes	yes	yes	yes
Q9: were additional interventions (co-interventions) clearly described?	no	no	no	no	no
Q10: were relevant outcome measures established a priori?	yes	yes	yes	yes	yes
Q11: were outcome assessors blinded to the intervention that patients received?	no	no	no	no	no
Q12: were the relevant outcomes measured using appropriate objective/subjective methods?	yes	yes	yes	yes	yes
Q13: were the relevant outcome measures made before and after the intervention?	no	no	no	no	no
Q14: were the statistical tests used to assess the relevant outcomes appropriate?	no	yes	yes	yes	yes
Q15: was follow-up long enough for important events and outcomes to occur?	yes	yes	yes	yes	yes
Q16: were losses to follow-up reported?	yes	no	no	no	no
Q17: did the study provided estimates of random variability in the data analysis of relevant outcomes?	no	yes	yes	yes	no
Q18: were the adverse events reported?	yes	yes	yes	no	no
Q19: were the conclusions of the study supported by results?	yes	yes	yes	yes	yes
Q20: were both competing interests and sources of support for the study reported?	no	no	yes	yes	yes
TOTAL (yes/no/unclear)	12/8/0	13/7/0	14/6/0	13/7/0	12/8/0

**Table 2:** Incidence of synchronous PJIs.

Study	Patients with Multiple Arthroplasties (*n*°)	Synchronous PJI (*n*°)	Percent
Zeller et al. [4]	1185	16	1.4%
Gausden et al. [5]	2671	34	1.3%
Thiensen et al. [6]	644	26	4%
Komnos et al. [7]	197	11	5%
Abblitt et al. [8]	76	4	5%

**Table 3:** Joints involved.

Study	Joints Involved
Zeller et al. [4]	8 bilateral THA, 3 bilateral TKA, 4 TKA and THA, 1 bilateral TKA + THA + toe arthroplasty
Gausden et al. [5]	27 bilateral TKA, 3 bilateral THA, 1 TKA + TSA, 1 TKA + TEA, 1 bilateral THA + TKA
Thiensen et al. [6]	20 THA, 15 TKA, 7 TSA
Komnos et al. [7]	19 THA, 4 TKA
Abblitt et al. [8]	3 bilateral TKA, 1 THA + TKA

Abbreviations: TKA, total knee arthroplasty; THA, total hip arthroplasty; TSA, total shoulder arthroplasty; TEA, total elbow arthroplasty.

**Table 4:** Bacteria associated with synchronous PJIs.

Study	*S. aureus* (*n*°)	*S. epidermidis* (*n*°)	*Streptococcus* spp. (*n*°)	*E. coli* (*n*°)	*P. mirabilis* (*n*°)	*N. meningitidis* (*n*°)	*Enterococcus* spp. (*n*°)	*R. Ornithinolytica* (*n*°)	*M. Chelonae* (*n*°)	Unknown (*n*°)
Zeller et al. [4]	8 (50%)	1 (6%)	6 (38%)	1 (6%)	/	/	/	/	/	/
Gausden et al. [5]	12 (35%)	1 (3%)	4 (12%)	1 (3%)	1 (3%)	1 (3%)	/	1 (3%)	1 (3%)	12 (35%)
Thiensen et al. [6]	5 (19.2%)	9 (34.6%)	2 (7.7%)	2 (7.7%)	/	/	3 (11.5%)	/	/	4 (11.5%)
Komnos et al. [7]	4 (36%)	3 (27%)	1 (9%)	1 (9%)	/	/	/	/	/	2 (18%)
Abblitt et al. [8]	3 (75%)	/	1 (25%)	/	/	/	/	/	/	/

**Table 5:** Risk factors for synchronous PJI.

Study	Risk Factors for Synchronous PJI
Zeller et al. [4]	Staphylococcal or streptococcal bacteremia
Gausden et al. [5]	Bacteremia
Thiensen et al. [6]	3 or more prosthetic joints, rheumatoid arthritis, neoplasia, immune-modulating therapy, bacteremia, sepsis
Komnos et al. [7]	Bacteremia
Abblitt et al. [8]	Bacteremia
Jafari et al. [10]	Immunosuppression
Luessenhop et al. [11]	Rheumatoid arthritis
Haverstock et al. [12]	Bacteremia

## Data Availability

The data reported in this study are available in the literature.
